# Gastroesophageal Junction Adenocarcinoma With Skeletal Muscle Metastases: A Case Report and Literature Review

**DOI:** 10.7759/cureus.63855

**Published:** 2024-07-04

**Authors:** Jacob Sabu, Aye M Thida, Gyuhee Seong, Amena Mohiuddin, Hagar Attia, Maksim Agaronov, Edwin Chiu

**Affiliations:** 1 Department of Medicine, State University of New York (SUNY) Downstate Health Sciences University/Kings County Hospital, New York, USA; 2 Department of Hematology and Oncology, State University of New York (SUNY) Downstate Health Sciences University/Kings County Hospital, New York, USA; 3 Department of Pathology and Laboratory Medicine, State University of New York (SUNY) Downstate Health Sciences University/Kings County Hospital, New York, USA

**Keywords:** rare metastasis, esophagus adenocarcinoma, gastroesophageal adenocarcinoma, ac-adenocarcinoma, muscle metastasis, skeletal muscle metastasis, gastroesophageal cancer, gastroesophageal junction (gej), esophageal cancer (ec)

## Abstract

Esophageal and gastroesophageal junction (GEJ) malignancies are aggressive, and survival is poor once metastasis occurs. The most common sites of metastatic involvement include the liver, lymph nodes, lung, peritoneum, adrenal glands, bone, and brain, while skeletal muscle (SM) involvement is rare. We report a case of a 68-year-old female who presented with intractable emesis for one month and was found to have a primary GEJ adenocarcinoma measuring up to 6.7 cm. Endoscopic biopsy revealed poorly differentiated GEJ adenocarcinoma with positive AE1/AE3 immunostains. Positron emission tomography/computed tomography and magnetic resonance imaging revealed metastases to the omentum and left lower extremity SMs, including the proximal adductor longus, adductor magnus, and gluteus minimus. This study reviews the literature on SM metastasis in esophageal and GEJ cancer, GEJ cancer classification, incidence, treatment, and prognosis.

## Introduction

Survival becomes increasingly challenging once metastasis occurs in esophageal and gastroesophageal junction (GEJ) malignancies, which are known for their aggressive nature. In the United States, the five-year overall relative survival rate from 2013 to 2019 was 21.7%, but survival with distant metastases was notably lower at 5.6% [1]. The most common sites for metastasis of both esophageal and GEJ tumors include the liver, distant lymph nodes, lung, peritoneum, adrenal glands, bone, and brain [2]. Skeletal muscle (SM) involvement in esophageal cancer is very rare [3,4], comprising approximately 9% of metastatic sites in the literature up to 2017 [2]. The researchers reviewed the literature to identify detailed case reports and case series of SM involvement in esophageal and GEJ cancers. There were only 16 detailed reports of esophageal cancer with SM metastasis in the English literature between 1986 and 2018, and only four such reports from these were of GEJ cancer between 2002 and 2016. We report the case of a 68-year-old female who presented with primary GEJ adenocarcinoma with this rare skeletal muscle metastasis (SMM).

## Case presentation

**Figure 1:**
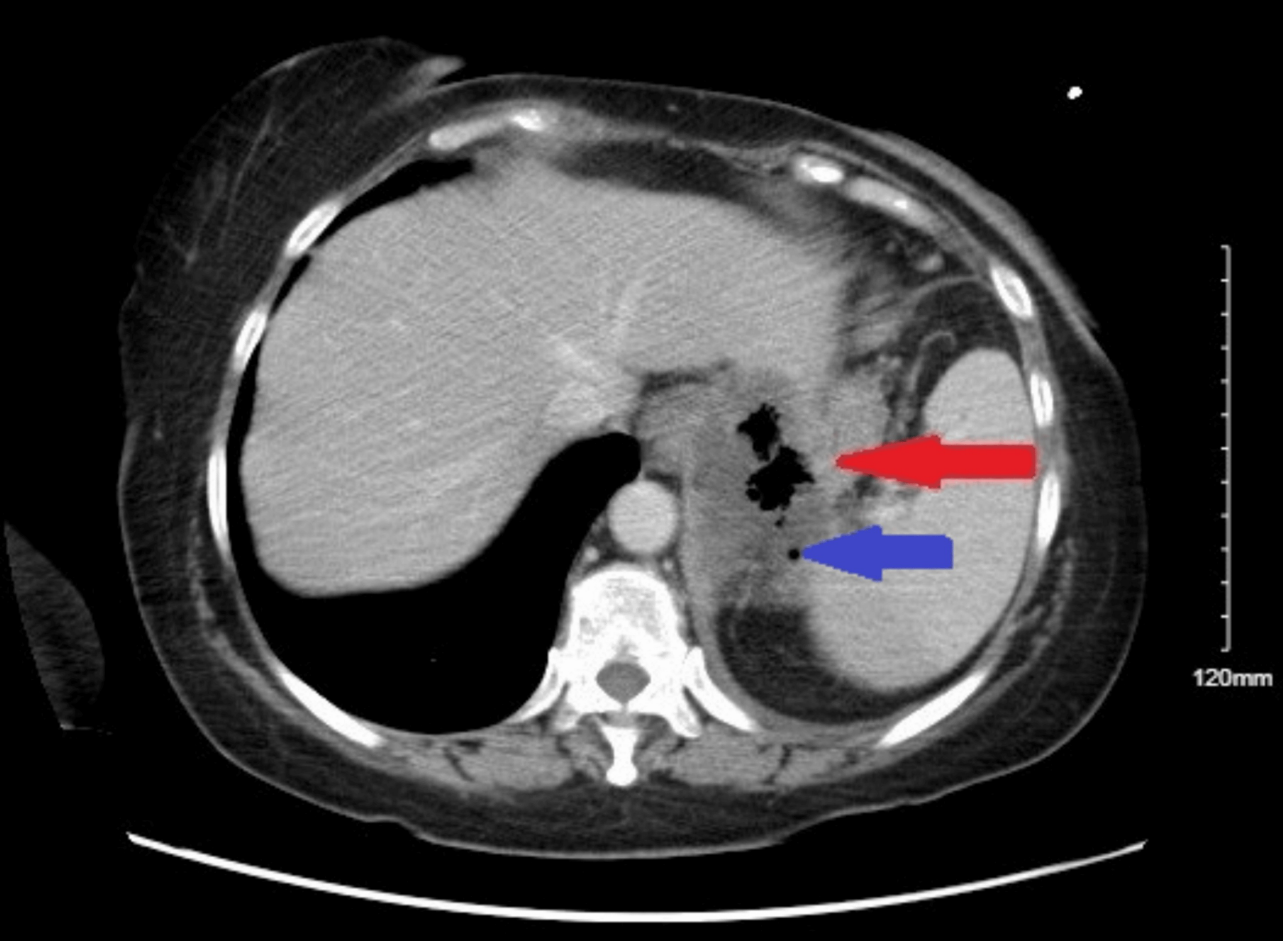
CT abdomen/pelvis with contrast showing a GEJ mass. GEJ mass measuring up to 6.7 cm (red arrow). Distal gastric fundus ulceration is adjacent to the GEJ, with concern for perforation (blue arrow) CT: computed tomography; GEJ: gastroesophageal junction

A 68-year-old female with a history of hypertension, diabetes mellitus, and hyperlipidemia presented with intractable emesis for a month. She felt that solid food was getting stuck in her chest and had to vomit after eating or drinking. She was frail-appearing and not acutely distressed; cardiopulmonary and abdominal examinations were unremarkable. Workup with computed tomography (CT) abdomen/pelvis with contrast revealed a GEJ mass measuring up to 6.7 cm, distal gastric fundus ulceration adjacent to the GEJ with concern of perforation, and thrombosis of the splenic vein and left adrenal vein (Figure 1).

**Figure 2:**
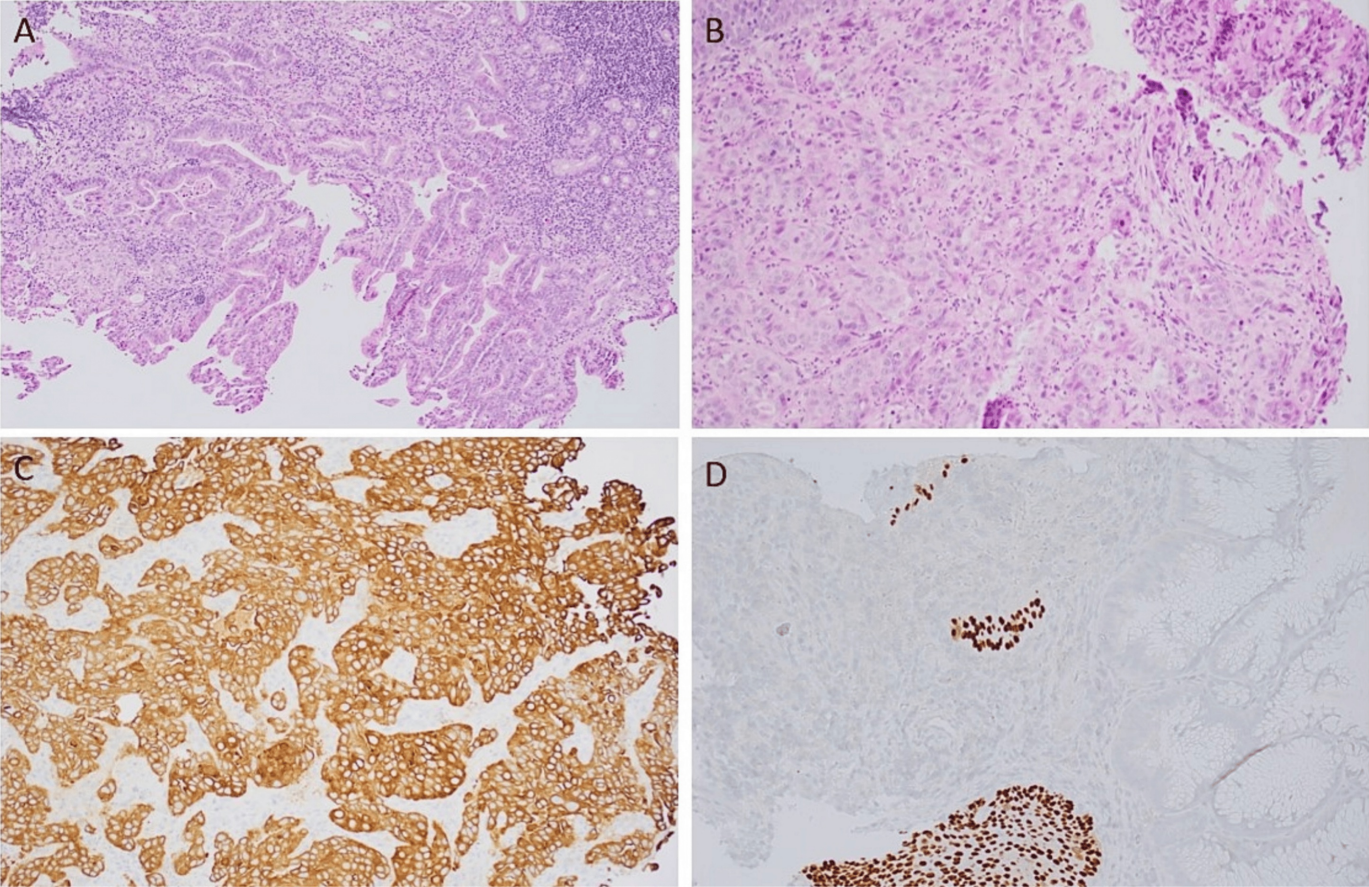
GEJ mass biopsy. (A) Hematoxylin and eosin, original magnifications 100×. (B) Hematoxylin and eosin, original magnifications 200×, showing moderately to poorly formed malignant glands. (C) The glands are positive for cytokeratin AE1/AE3 on IHC. (D) The glands are negative for p40 on IHC GEJ: gastroesophageal junction; IHC: immunohistochemistry

A follow-up esophagram was performed with Gastrografin, and there was no demonstration of perforation. During the esophagogastroduodenoscopy, a partially obstructing, likely malignant tumor was found at the GEJ and in the cardia, which was biopsied. The endoscopic ultrasound of the tumor was deferred due to concern about perforation. Histologic examination of the distal esophagus mass demonstrated moderately to poorly differentiated adenocarcinoma, with positive cytokeratin AE1/AE3 immunohistochemistry (IHC) and negative for p40; IHC for human epidermal growth factor receptor 2 was negative (Figure 2). The glands are negative for p40 IHC, which helps rule out squamous cell carcinoma. The positive background staining of the benign esophageal squamous mucosa is notable (Figure 2D).

**Figure 3:**
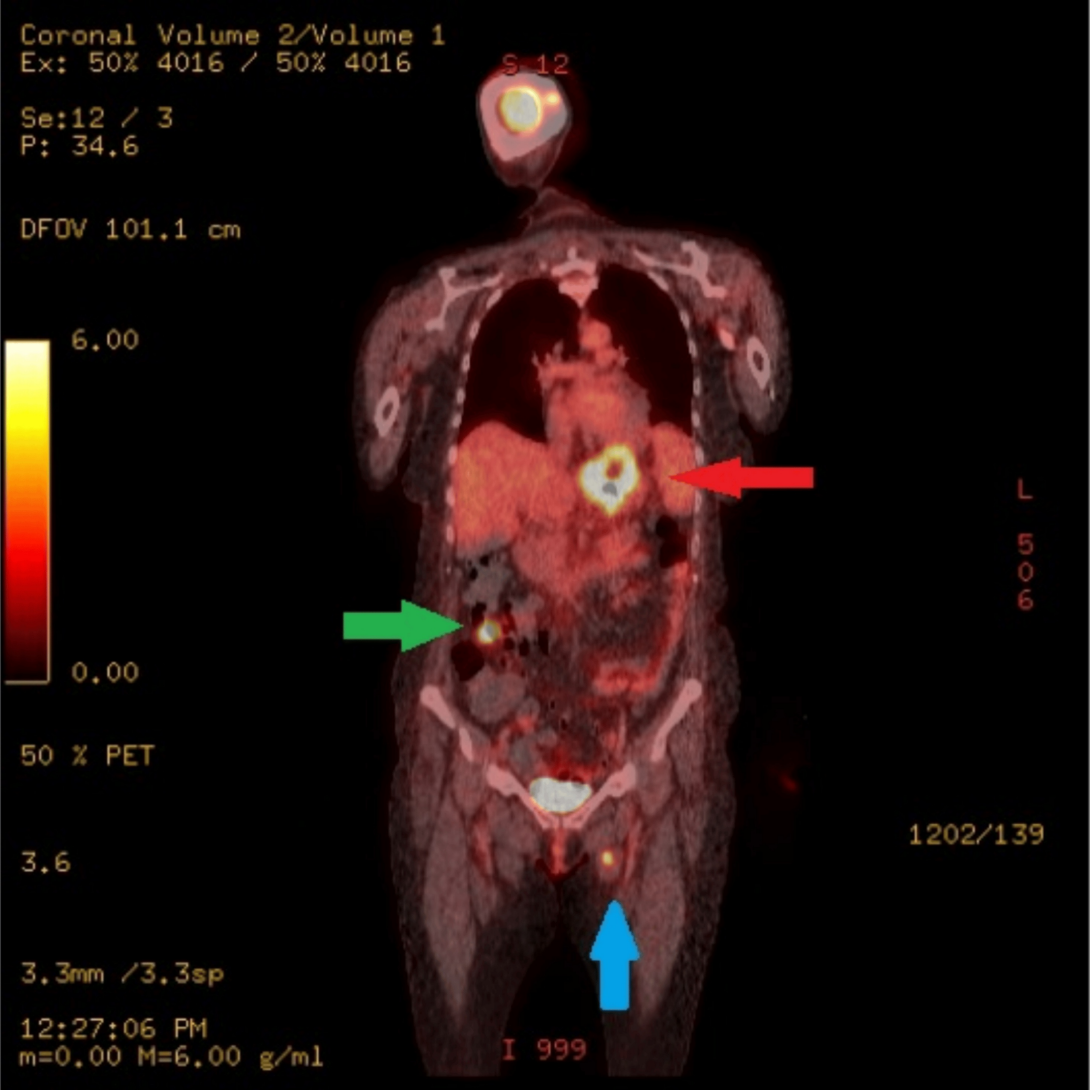
PET/CT. FDG avid necrotic distal esophagus and gastric fundus lesion (red arrow), SUV max 14, consistent with biopsy-proven adenocarcinoma of the esophagus. Focal FDG avidity along the proximal left adductor muscle (blue arrow), SUV max 6.49, representing intramuscular metastasis. FDG avid left adrenal nodule measuring up to 2 x 1.5 cm, SUV max 7.02, likely metastatic in etiology. FDG avid right lower quadrant peritoneal soft-tissue attenuation lesion measuring 2.4 x 1.8 cm, SUV max 8.7, likely reflective of peritoneal metastasis (green arrow) PET/CT: positron emission tomography/computed tomography; FDG: fludeoxyglucose F18; SUV max: maximum standardized uptake value

Positron emission tomography (PET)/CT showed hypermetabolic activity with suspicion of metastatic disease or microperforation (Figure 3).

**Figure 4:**
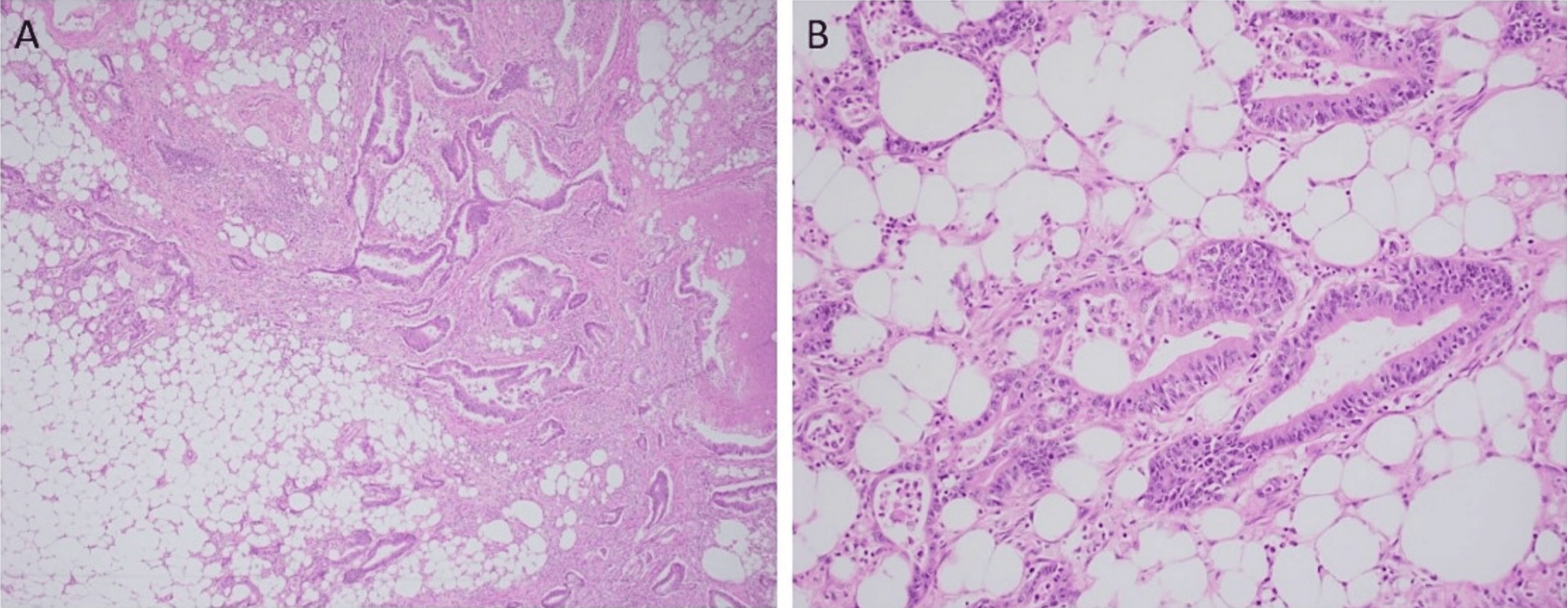
Omental biopsy. (A) Hematoxylin and eosin, original magnifications 40×. (B) Hematoxylin and eosin, original magnifications 200×, exhibiting infiltration of the omental fat by malignant glands indicative of metastatic adenocarcinoma from the GEJ mass GEJ: gastroesophageal junction

A diagnostic laparoscopy was performed, and a small omental implant on the right side was biopsied. Later, it was confirmed to be metastatic adenocarcinoma morphologically similar to the esophageal tumor (Figure 4). There was no gross peritoneal carcinomatosis.

**Figure 5:**
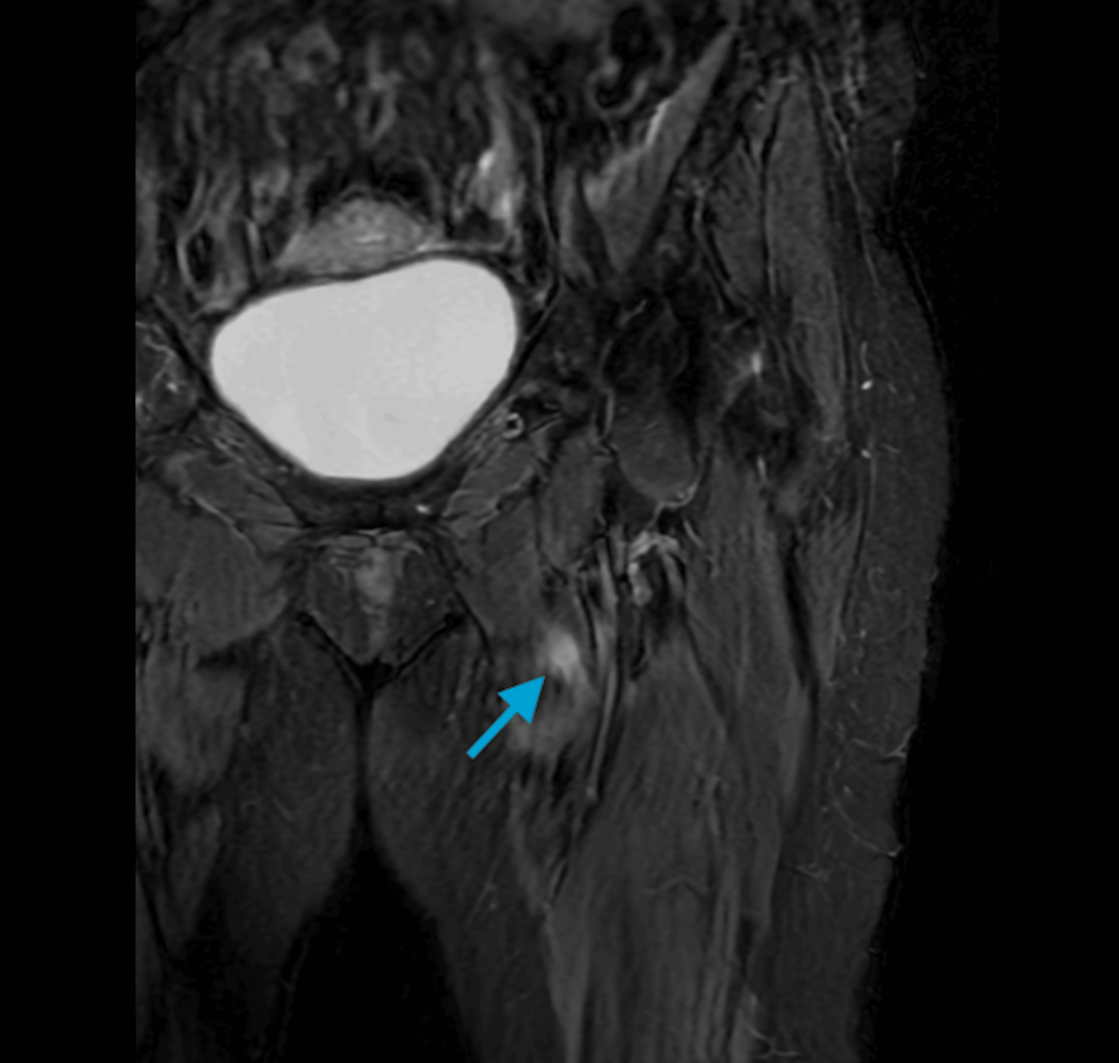
MRI of the left femur with contrast demonstrating a 1.2 x 1.2 x 2.5 cm enhancing lesion within the proximal left adductor longus muscle, which corresponded to the hypermetabolic focus seen on PET/CT scan (blue arrow) MRI: magnetic resonance imaging; PET/CT: positron emission tomography/computed tomography

Magnetic resonance imaging (MRI) of the left femur showed a 1.2 x 1.2 x 2.5 cm enhancing lesion within the proximal left adductor longus muscle, which corresponded to the focus seen on PET/CT (Figure 5).

**Figure 6:**
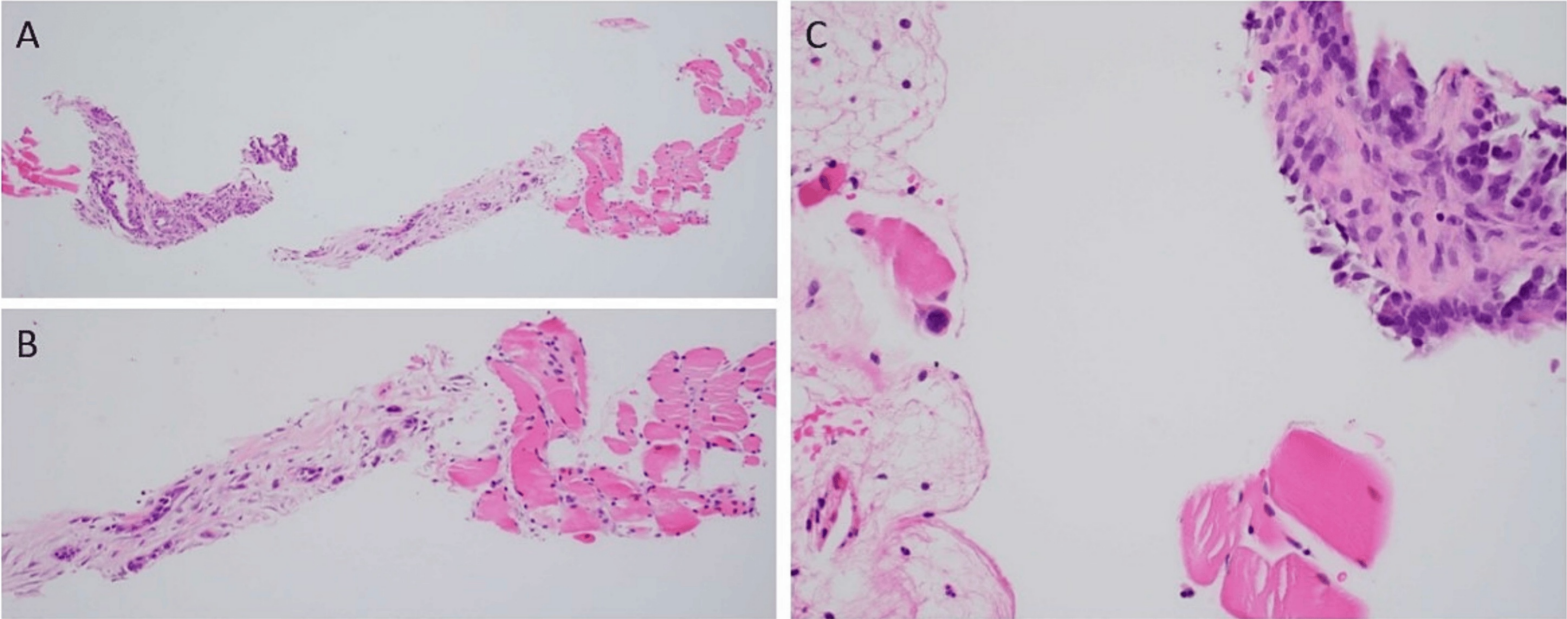
SM biopsy. (A) Hematoxylin and eosin, original magnifications 100×. (B) Hematoxylin and eosin, original magnifications 200×. (C) Hematoxylin and eosin, original magnifications 400×: biopsy of the muscle involved by metastatic adenocarcinoma with infiltrative moderately to poorly formed malignant glands SM: skeletal muscle

Multiple smaller enhancing lesions were seen within the adductor magnus and gluteus minimus muscles in the MRI of the femur, with a biopsy of the involved muscle revealing adenocarcinoma (Figure 6).

The case was discussed in the multidisciplinary tumor board conference, and the consensus was to give palliative radiation therapy for local control of impending perforation at the tumor site. It was concluded that administering chemotherapy in cases of contained or potential tumor perforation carries a significant level of risk. However, following the patient’s discharge from the hospital, outpatient palliative radiotherapy was unattainable due to the patient's illness.

A month after discharge, the patient presented with intractable emesis, inability to tolerate oral intake, and abdominal pain. The CT scan of the abdomen and pelvis showed the presence of free air under the diaphragm, suggestive of perforation, and an ulcerated gastric fundal mass with an enlarging left subphrenic irregular phlegmon measuring approximately 6 cm extending directly into the spleen. The patient was admitted for failure to thrive and sepsis due to possible aspiration pneumonia and phlegmon. The patient was deemed not a candidate for palliative surgery or radiation due to her frail condition. The case was discussed in a multidisciplinary tumor board conference with a consensus on the best supportive care. A palliative esophageal stent was placed due to complete obstruction caused by the esophageal mass. The patient expired after four months after the initial diagnosis.

## Discussion

Definition and classification of GEJ cancer

Gastric and esophageal cancers include esophageal squamous cell carcinoma, proximal GEJ adenocarcinomas (in the esophagus and gastric cardia), and distal gastric adenocarcinoma. The vast majority of these tumors are histologically adenocarcinomas.

GEJ tumors are classified into three different subtypes by the Siewert classification, developed by the German surgeon Jörg Rüdiger Siewert. Siewert type I GEJ tumors are between 1 and 5 cm proximal to the GEJ. Siewert type II tumors cross the anatomic GEJ with their epicenter up to 2 cm below GEJ, and Siewert type III tumors span between 2 and 5 cm into the stomach [5]. The Siewert classification has been broadly used to determine surgical approaches for GEJ tumors. Siewert type I GEJ tumors are treated as esophageal cancer requiring partial gastrectomy, while type III GEJ tumors are treated as gastric cancer requiring total gastrectomy according to the consensus from randomized controlled trials [6]. Siewert type II GEJ tumors are treated as esophageal cancer, similar to type I, but with differing regions of mediastinal lymph nodes to be resected based on tumor invasion [6]. All three Siewert types require abdominal lymph node resection, with increasing amounts to be resected with increasing Siewert grade [6]. Our patient presented with a type III GEJ tumor due to its distension into the gastric cardia. However, due to the presence of metastases, systemic chemotherapy would have been required rather than the proposed surgical resection and/or radiotherapy.

Metastasis of GEJ cancers in the SM

SM involvement in GEJ cancers is rare [3,4]. We reviewed the literature on SMM from esophageal and GEJ tumors reported in PubMed between 1986 and 2024 (Tables 1, 2).

**Table 1:** Esophageal cancer with SMM ND: not described; SCC: squamous cell carcinoma; L: left; adeno: adenocarcinoma; LNs: lymph nodes; 5-FU: 5-fluorouracil; R: right; TPF: docetaxel (Taxotere), cisplatin (Platinol), and 5-fluorouracil; SCC: squamous cell carcinoma; FOLFOX: leucovorin calcium (folinic acid), fluorouracil, and oxaliplatin; SMM: skeletal muscle metastasis

Authors	Year	Age (years)	Sex	Primary histology	Affected muscle	Other metastases	Therapy	Outcome
Schultz et al. [7]	1986	ND	ND	SCC	L gluteus minimus	ND	ND	ND
Heyer et al. [8]	2005	54	M	Adeno	Erector spinae, external obliques, internal obliques, rectus abdominis, latissimus dorsi, gluteus maximus, gluteus medius, biceps femoris, quadriceps femoris, semimembranosus, and semitendinosus	LNs around celiac artery	Radiation, 5-FU, and cisplatin	ND
Heffernan et al. [9]	2006	67	F	Adeno	R infraspinatus, R obturator externus, and L gluteus medius	Mediastinum, R external iliac LN	ND	ND
Kozyreva et al. [10]	2007	72	M	Signet ring adeno	L deltoid, R gluteus maximus, L gluteus maximus, L sartorius, and mid-dorsal paraspinals	None found	Floxuridine, leucovorin, oxaliplatin, and docetaxel	Tumor reduction
Norris et al. [3]	2009	58	M	Adeno	R ilium and R iliacus	Liver, adrenals, and para-aortic and aortocaval LNs	ND	ND
Uygur et al. [11]	2011	62	F	Basaloid SCC	Temporalis	ND	Temporalis tumor resection	ND
Cincibuch et al. [4]	2012	64	M	Adeno	L quadriceps femoris	Bone and LNs	Radiation, 5-FU, and cisplatin	Deceased 31 months after muscle metastasis
		76	M	SCC	R gluteus minimus and L psoas	Lungs, pleura, adrenals, and LNs	Radiation, 5-FU, and cisplatin	Deceased 16 months after muscle metastasis
		57	M	SCC	R subscapularis	Bone	5-FU, cisplatin, and palliative radiation	Deceased five months after muscle metastasis
		42	M	Adeno	Iliacus	Peritoneum	Radiation, 5-FU, cisplatin, esophagectomy, and met resection	Progression of peritoneal carcinomatosis. Later deceased 16 months after muscle metastasis
		60	M	SCC	Multiple muscles	Liver, left adrenal, bone, and LNs	Palliative	Widespread metastatic progression. Later deceased two months after presentation
Leuzzi et al. [12]	2013	65	M	Adeno	R lumbar paravertebrals	None found	Chemotherapy and esophagectomy	ND
Sohda et al. [13]	2014	49	M	Adeno	L thigh	Paraesophageal and R cardiac LNs	5-FU, nedaplatin, and, later, TPF	Metastatic progression. Later deceased six months after initial treatment
Saito et al. [14]	2017	56	M	SCC	L shoulder	Cervical and mediastinal LNs, and stomach	5-FU, nedaplatin, and, later, S-1 fluoropyrimidine	No recurrence after initial treatment
Fujimoto et al. [15]	2017	77	M	SCC	L extensor digitorum communis, L abductor pollicis longus, and L extensor carpi ulnaris	None found	Radiation, 5-FU, and cisplatin	Complete response maintained 20 months after initial treatment
Thumallapally et al. [16]	2018	73	M	Adeno	L medial rectus	Lungs, appendicular skeleton	FOLFOX, paclitaxel, ramucirumab, and palliative radiotherapy	Deceased from sepsis

**Table 2:** GEJ cancer with SMM GEJ: gastroesophageal junction; R: right; ND: not described; 5-FU: 5-fluorouracil; adeno: adenocarcinoma; SMM: skeletal muscle metastasis

Authors	Year	Age (years)	Sex	Primary histology	Affected muscle	Other metastases	Therapy	Outcome
Rehman et al. [17]	2002	71	M	GEJ adeno	R thigh	ND	Radiation	Symptomatic relief maintained for two weeks
Lekse et al. [18]	2003	78	F	GEJ signet ring adeno	R orbital inferior rectus	Lung	Hospice	ND
		78	M	GEJ adeno	R orbital lateral rectus	Bone, lung, and liver	5-FU and leucovorin	ND
Wu et al. [19]	2005	67	M	GEJ adeno	R gluteus minimus	ND	ND	ND
Azadeh et al. [20]	2016	65	M	GEJ adeno	R psoas	None found	Oxaliplatin, capecitabine, Paclitaxel, carboplatin, and radiation	Complete response maintained to time of publication
Our study	2024	68	F	GEJ adeno	Adductor longus, adductor magnus, and gluteus minimus	Omentum	Palliative	Deceased four months after initial diagnosis

Even though SM comprises 50% of the body mass and is highly vascularized, SMM is uncommon in patients with GEJ metastatic disease, with less than 10% of patients having metastatic involvement limited to SM [2]. The rarity of these metastases can be attributed to various factors. These include the variability of blood flow in muscles during rest and exercise, which reduces the adherence of tumor cells to the surrounding tissues. SMs also produce substances like leukemia inhibitory factor and interleukin-6 that have anticancer properties [21]. Additionally, the microvasculature of actively contracting SMs may have the ability to biomechanically destroy cancer cells, which is in contrast to denervated and noncontracting muscle in which cancer cell survival is relatively greater [22].

Muscle metastasis poses a diagnostic challenge because it is usually asymptomatic. To detect SM involvement, PET/CT imaging is widely used to stage esophageal carcinoma and can detect distant metastases that may be missed by other imaging studies [7-9]. SMM cases have been identified in patients staged with PET/CT, suggesting that the increased incidence of SMM in esophageal carcinoma may be due to improved diagnostic capabilities and better control of the primary tumor through combined treatment approaches. A diagnostic biopsy is required for confirmation.

There is limited knowledge about the outcomes and optimal management strategies for patients with SMM in GEJ metastasis, and most of the information available comes from anecdotal reports. General recommendations for managing SMM, including SMM of esophageal carcinoma, focus on symptom control and tailoring the therapeutic approach based on the affected muscle. Surgery may be the preferred option for isolated SMM, as it can provide the best control and also help establish the diagnosis. External beam radiation may be used for inoperable or recurrent SMM, especially when combined with platinum-based chemotherapy [4]. The role of systemic chemotherapy in managing SMM appears to follow the general GEJ metastasis treatment guideline, but it may be considered in patients with widespread metastases and good general condition [23].

## Conclusions

In this article, a case of SMM of primary GEJ cancer is reported, and the literature on SMM in GEJ cancer is reviewed. A confirmatory biopsy is required for diagnosis. Given the limited number of reported cases of SMM, it is challenging to generalize prognostic effects and outcomes. With the increased incidence of GEJ adenocarcinomas in Western countries from improved imaging, it would be crucial to diagnose and treat GEJ adenocarcinomas effectively. Further studies of SMM from upper GI origin will elucidate the prognosis and treatment of GEJ cancers.
